# Ethnotaxonomy of mastofauna as practised by hunters of the municipality of Paulista, state of Paraíba-Brazil

**DOI:** 10.1186/1746-4269-2-19

**Published:** 2006-04-07

**Authors:** José S Mourão, Helder FP Araujo, Fabiana S Almeida

**Affiliations:** 1 Departamento de Biologia, Núcleo de Etnoecologia, Educação Ambiental e Gestão, Universidade Estadual da Paraíba. Campus Universitário do Bodocongó, Campina Grande, PB, Brazil; 2 Programa de Pós-Graduação em Ciências Biológicas (Zoologia), Departamento de Sistemática e Ecologia, Universidade Federal da Paraíba, 58051-900 João Pessoa, PB, Brazil; 3 Prefeitura de Serra Negra, RN, Brasil

## Abstract

It was aimed in the present work to report aspects related to identification, naming and categorization of the mastofauna species of the caatinga biome, according to hunters' knowledge of Northeast Brazil. The methods of free and semi-structured interviews and guided tours were applied here. The data obtained were analyzed under the emic/etic point of view by comparing the local people's knowledge to those reported in the literature. The inland hunters use some classification models of mammals living around them as for example the folk taxonomy, and a categorization based on the animal utility. Some species are preferably hunted for providing food while others are hunted for therapeutic ends. Hunt techniques were made evident by the informers. The retrieval and comprehension of the whole process related to such knowledge is very important to evaluate the human culture and the protection that people exert on local biodiversity, since these aspects have implications for the conservation and management of faunistic resources.

## Introduction

The knowledge on natural world that peoples hand down orally through the generations are usually denominated as 'local' or 'traditional'. This knowledge is culturally transmitted through the generations by using symbols, phonetic, narratives, rituals, music, and dance [1], which are imprinted in people's mind as memes [2]. There is a variety of terms describing traditional knowledge and practices, *e.g. *the indigenous technical knowledge (ITK), traditional ecological knowledge (TEK), rural people knowledge (RPK), traditional botanical knowledge (TBK), and integrated knowledge system (IKS) [3].

In the last years, traditional populations' wisdom has been deemed as extremely important for the development of studies in several areas of natural sciences. This knowledge is the aim of ethnoscience studies, where the prefix *ethno *refers to the system of knowledge and cognition that are typical of each culture [4].

Among the ethnosciences, the ethnobiology deserves special attention by involving the analysis of the classification system on Nature and for having a deep link with the subjects of botany, zoology, and ecology. Lévi-Strauss was one of the forerunners in this field of study, mainly for the emphasis he gave to the classification systems allowing to reach the logic of the native thought [5].

There have been great contributions of ethnobiological studies to the academic advancement of biology, which can be illustrated as follows: (i) nine species of non-sting bees (Meliponinae) were discovered thanks to the Kayapó Indian's ethnoentomology (in the Amazon basin) [6]; (ii) the number of fishes forming lunar reproductive aggregates, which are known to biologists, has more than doubled as a consequence of ethnoichthyology of fishermen from the Pacific islands [7]; (iii) the feeding behaviour of marmosets of the genus *Callithrix *was evaluated with the help of woodmen's ethnoethology of the State of Alagoas, Northeast Brazil [8,9].

Human being/animal is a quite ancient relation and an extremely important connection to human societies, because the latter depends very often on the former for their survival. Ethnozoology was the name given to this subfield of work by Mason in USA, in 1899, who defined it as 'the zoology of the region as actually told by the indigenous" [10]. Ethnozoology, as part of ethnobiology, is concerned with the revelations of traditional populations' knowledge on wildlife, as well as their reciprocal relationship that is aimed at understanding the role of animals in the daily lives of human communities [9].

The biodiversity of the caatinga, (vegetation consisting of thorny shrubs and stunted trees situated in the interland of Northeast Brazil) have long been deemed as rather low [10-12]. However, recent studies revealed a number of animals expressively higher than the formerly reported ones, tearing down the myth of the poor caatinga's biodiversity [13-15]. The figures currently reported, however, may even be increased, since 41% of the entire caatinga area [larger than 800,000 km^2 ^in Northeast Brazil] has never been scientifically investigated, and 80% of the studied areas are rather small representative sampling effort [16].

The dwelling places of inland herdsmen of the state of Paraíba extend from the 'agreste' (a transitional microregion between the littoral woods and the caatinga ecosystems of the 'carirí', a quite semi-arid microregion) to 'sertão' (the caatinga ecosystems microregion on the northwest) [17]. Such people grow crops and raise cattle, besides carrying out hunting activities as means of subsistence. The inland dweller is primarily a hunter, fisherman, or grower, and only after that, he is able to carry out another task [18]. Despite the northeastern Brazilian region be a vast field for ethnobiological studies, the inlander's behaviour exploiting the natural resources has not been much investigated [17].

It was aimed in the present work to obtain information about the regional mastofauna of Paraiban caatingas at the municipality of Paulista and its use by local inland herdsmen.

### The study area

**Figure 1:**
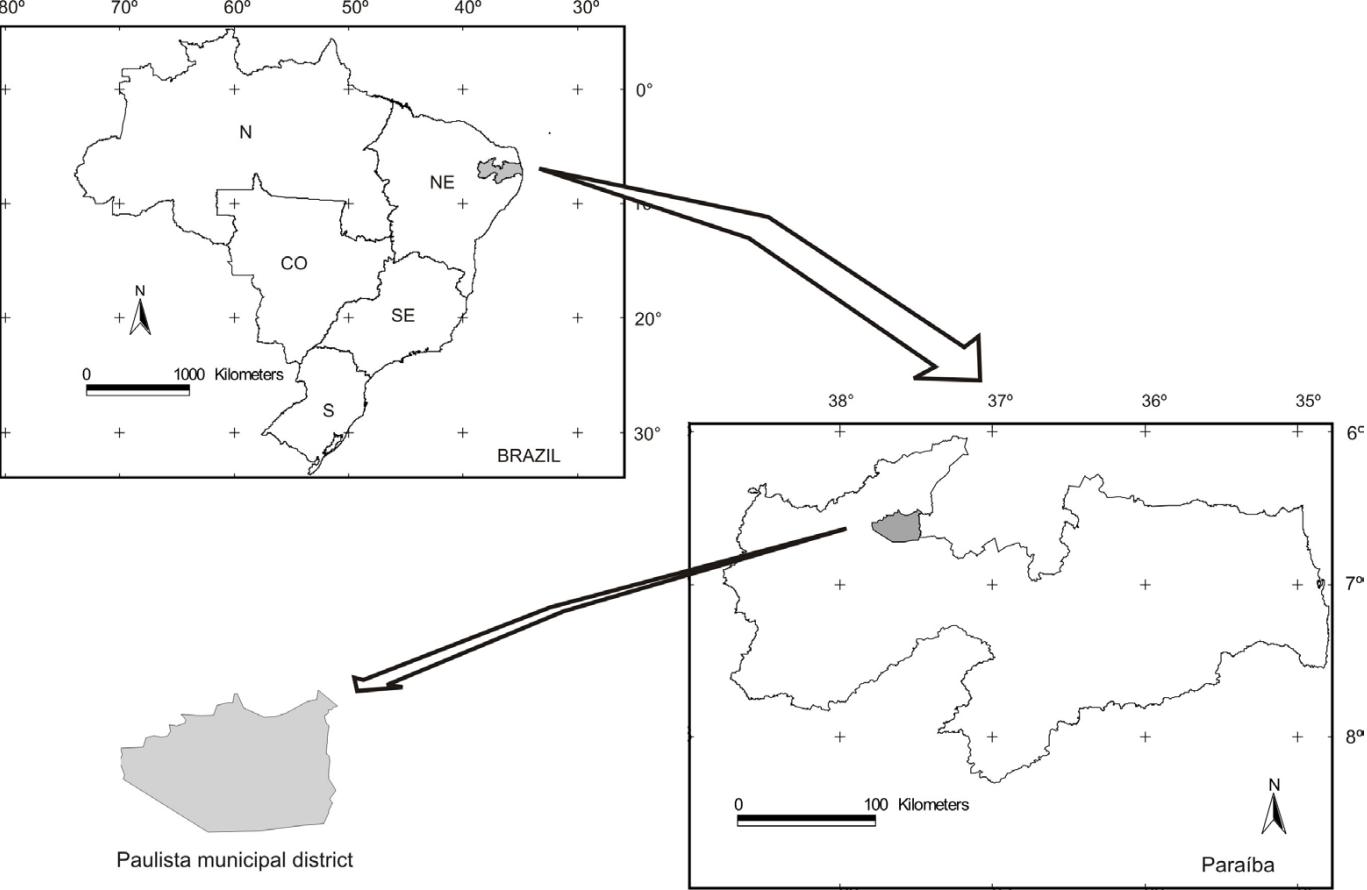
Localization of the municipality of Paulista, in the caatinga region of the state of Paraíba, Northeast- Brazil.

The municipality of Paulista is situated in a caatinga region designated as septentrional inland depression, whose geographic coordinates are at lat 6°35'58"S and long 37°37'27"W and at an altitude of 160 m (Figure 1). The Piranhas River, a permanent watercourse, is the main hydrologic resource of the municipality of Paulista.

The climate of the region is typically semi-arid, with an average annual rainfall of 400 mm. The population of that municipality is of 15,349 [19].

The natural environment, the caatinga, has the ground covered with herbaceous but non-grassy plants and the predominance of tropical xerophytes, which are tortuous thorny shrubs and stunted trees, having small and caducous leaves [18].

### Methodological procedures

We performed 20 free and open interviews, in Portuguese language, with local hunters, inland herdsmen, in period of 2003 and 2004. The hunters has a low literary degree, however know to read and write.

We asked about the mastofaunistic composition known to them, followed by semi-structured direct forms on specific topics of the mastofauna, as nomination, identification and use [20-22].

Only the most experienced hunters were interviewed. Their own choice followed the 'snow ball' technique, that consists of the indication in sequence of recognized informers, exactly, for the degree of experience [23,24].

The names of mammals known to the informers were obtained from them by applying the free listing technique, that looks specific informations on a cultural domain of the studied community. The hunters were requested to list the names of known mammals [21]. The limitations of free listing technique were overcome by adopting the 'non-specific prompting' and 'reading back' techniques. The first consists of questions made to the informers when they declare not to remember of more elements, however those inductions should be sentences formulated that do not generate answers yes or not. The second is a technique used soon after, where the researcher reads the supplied list slowly and allowed the citation, by informers, of items not listed previously [21,25].

The morphological characteristics of some mammals they mentioned were obtained from the informers with the help of photographs and cards containing the animal's images, the scientific correspondents follow the nomenclature of the list of the Caatinga biome [26].

In ethnotaxonomy, recongnized ethnobiological taxa are taxonomically distributed as members of six mutually exclusive ethonobiological ranks comparable in content to the ranks of Western zoology and botany [27]. The six ranks, in descending order of taxonomic inclusiveness, are the kingdom, life-form, intermediate, generic specific and varietal. The rank of kingdom is unique in that it but a single member. For ethnobiologically, the kingdom corresponds approximately to the biological taxons Plantae and Animalia. Taxa of the life-form mark a small number of highly distinctive morphotypes based on the recognition of the strong correlation of gross morphological structure and ecological adaptation. Taxa of intermediate rank are found most commonly as members of life-form taxa, and are comprised of small numbers of folk generics that show marked perceptual similarities with one another. The most numerous taxa in folk biological taxonomies will be taxa of generic rank. Roughly 80 percent of folk generic taxa in tipical folk systems are monotipic and include no taxa of lesser rank. Taxa of the rank of folk species partition folk generic taxa into two or more members; in those systems where they occur, folk varietals further subdivide folk species.

Some nominations of generics are not classified in hierarchical structure. However, they have a relation with cultural and/or economical importance. Some generic mentioned were also classified in an utilitarian way.

## Results and discussion

The hunters' information totalled 20 genera of the mastofauna (Table I), which comprise 20 species of mammals out of 148 known species of the caatinga biome [15,24].

**Table 1:** The generic folk of the mastofauna mentioned by the hunters of the municipality of Paulista, State of Paraíba, showing their respective scientific names, local habitats, and the habitats reported in the literature: 1 39 and 2 40

		Habitats of the mammals		
Generic folk	Corresponding taxon	Macrohabitat	Microhabitat	Habitats/[literature]
	**Artiodactyla **Tayassuidae			
Collared peccary	*Pecari tajacu*	.....Rocky hill	Den	Den/[2]
	**Carnivora **Canidae			
Crab-eating fox	*Cerdocyon thous*	Sertão	Burrow and den	Thickets, tablelands/[2]
Jaguarundi	Felididae *Herpailurus yaguarondi*	Everywhere	Den and tree	Rocky hill and tablelands/[1]
Margay	*Leopardus ........wiedii*	Everywhere	Den and tree	Rocky hill and tablelands [1]
Greater grison	Mustelidae *Galictis vittata *and/or *G. cuja*	Rocky hill	Burrow, den, tree	(No reference)
Crab-eaten raccoon 'Guará'	Procyonidae *Procyon cancrivorus *(Unidentified species)	'Island'* Rocky hill, 'island'	Den, tree Den	Thickets/[1] (No reference)
	**Didelphimorphia**			
Skunk and 'timbu' (opossums)	Didelphidae	Rocky hill, 'island'	Burrow, den tree	Rocky hills, trees/[1]
	**Primates **Callithrichidae			
Common marmoset	*Callithrix jacchus *Cebidae	Sertão	Slope, tree	Trees [2]
Tufted capuchin	*Cebus apella*	Rocky hill	Tree	Trees [2]
	**Rodentia **Caviidae			
Rock cavy	*Kerodon rupestris*	Rocky hill	Cracks in the stones	Caves, grottos, rocky hills/[1]
Spix's yellow- toothed cavy	*Galea spixii*	Everywhere	Den and crevices in the rocks	Rocky hills and grass fields/[1]
Rats	Muridae		Everywhere	(No reference)
	**Xenarthra **Dasypodidae			
Nine-banded armadillo	*Dasypus novemcinctus*	Rocky hill, 'island'	Burrow	Burrows in rocky hills/[1]
Six-banded armadillo	*Euphractus sexcinctus*	Rocky hill, 'island'	Burrow, den	Burrows in rocky hills/[1]
Giant anteater	Mymercophagidae *Mymercophaga tridactyla*	Rocky hill, 'island', Woods	Den, tree	(No reference)
Southern tamandua	*Tamandua tetradactyla*	Rocky hill, 'island', Woods	Den, tree	(No reference)

* 'Island': a lowland area surrounded by stagnant water

The hunters we interviewed have got a detailed knowledge of the mastofauna resource of caatinga ecosystems, which enables them to show a folk taxonomy through identification, denomination, and classification of organisms. This way, they utilize morphological, ecological, and behavioural criteria. Their ability for passing on information on the habitat, trophic relations, behaviour, and morphological details of animals, is a relevant feature of folk classification systems, an aspect also found among other Brazilian human groups reported elsewhere [27-36].

Biological classifications make up a general reference system on biological diversity and are actual storehouse of information [37]. In this sense, ethnobiological classifications are also a storehouse of information, since they contain immense richness of information on the biology, ecology, and ethology of several groups of animals and plants.

The ethnobiological classification system is conceptually organized as a shallow hierarchical structure [27]. Thus, hunters categorize mammals hierarchically, as we can observe from their expressions: '*there are two kinds of anteaters*'; '*there are many kinds of cats*'.

According to the hunters, a mammal is any sort of animal that has the habit of feeding its young on milk. This is a purely biological-based classification criterion, where the taxon of the life-form is known as animals that suck or that possess mammary glands. Despite they recognize this category as bearing similar characteristics, they also make evident that there is a great diversity of distinct kinds of mammals. The most related or similar kinds are generally grouped in one single category called 'family', as can be noticed from some parts of their interviews:

• '*I think that they are of the same family: skunk, anteater, and opossum are of a same similarity*'.

• '*The six- and nine-banded armadillos are quite alike: they must belong to the same group*'.

• '*Anteater and skunk have similar furs*'. '*I believe that the crab-eating raccoon and crab-eating fox are of a single family; of a single species*'.

• '*The cat, despite its different fur, is also an animal that feeds on the same kind of food that is preferred by the crab-eating raccoon and crab-eating fox*'.

• '*Monkey and marmoset are of the same species; some of them being larger than others*'.

Those groupings do not indicate hierarchical relations in taxonomy folk, but they are made with morphologic similarities and allow a comparison with the biological taxonomy. Within the scientific taxonomy, the rock cavy (*Kerodon rupestris*) and the Spix's yellow-toothed cavy (*Galea spixii*), and rats (several species of Muridae) are species of the same order Rodentia, which were grouped in the same ethnobiological taxon by the informers. The hunters also use another form for identifying the animals, through ecological criterion, *i.e. *their feeding habits, as in the generic folk cat (several species of Felidae) and the crab-eating raccoon (*Procyon cancrivorus*), which are carnivores (order Carnivora]. The semantic and ecological dimensions of animal identification show how important it is to recognize it for our comprehension on popular taxonomy [22]. Nominations that indicate hierarchical and no-hierarchical relations were also recorded in a study of classification ethnozoological of Nage, in western Indonesia [38].

Besides the hierarchical categorization, hunters use other classification systems. They classify mammals, for example, according to their utility (for human purposes): 'hunting animals' and just 'animals'. In the former category they include the nine-banded armadillo (*Dasypus novemcinctus*) and the six-banded armadillo (*Euphractus sexcinctus*) which are of the same generic folk and are the most hunted animals. Such classification criteria have also been used by other peoples of aural tradition, like the Montagnais ethnozoological classification [42], which categorize the animals as edible (red meat and white meat) and non-edible.

The inland hunters of the State of Paraíba identify and name mammals by taking in account their colour, body shape, size, or a typical characteristic of any part of the animal. As an example, the generic 'red cat' or margay (*Leopardus wiedii*) is so called because '*it is entirely red*'. They also justify naming some mammals for their analogy with objects, like the anteater called by them as 'tamanduá-bandeira' literally meaning 'flag anteater', because of its fury tail looking like a flag (a designation widely known in Brazil for this animal). Another anteater is called by the hunters as 'leather-tailed' anteater because of its naked tail, or as they say: '*it's smooth; it's really a long leathery tail*'. The same criteria they use for naming mammals are generally used for identifying them. Such identification criteria are purely morphological, like the ones used for the generic folk Spix's yellow-toothed cavy (*Galea spixii*) and the rock cavy (*Kerodon rupestris*), and also for rats (several species of Muridae), whose body shapes and existent or non-existent body parts and even their size are useful for their identification. The generic folk rat (Muridae) has got a tail that makes it distinct from the rock cavy (*K. rupestris*). This latter and the Spix's yellow-toothed cavy (*G. spixii*) differ with respect to body length, the red hind back of the former and also with respect to their habitats; the informers say these are the most conspicuous features of each generic folk. The animals' habitats in the caatinga are classified by the hunters as 'macrohabitats', like (i) rocky hills, (ii) sertão, a plain covered by the native vegetation, and (iii) 'island', a lowland area surrounded by stagnant water; and 'microhabitats', like (a) burrow, (b) den, among rocks, (c) cave, (d) tree, (e) slopes, (f) tree hole, and (g) dark hiding places. In Table I the habitats of the generic terms mentioned here are presented, in comparison with other denominations reported elsewhere. This kind of habitat categorization, also used for identifying organisms concerning their distribution and the areas they share, has been widely reported in the ethnoecological literature on several human populations [8,42-44].

In the same way of the generic folk above-mentioned, the informers say that the crab-eating raccoon (*P. carnivorus*) and the 'guará', an unidentified species of the family Canidae, differ with respect to size and colour: this latter is a smaller and more dark-coloured animal. The generic 'ticaca' and 'timbu' (both species are some kind of opossum, family Didelphidae) differ by their morphology, like type and coloration of fur, as said by one of the informers: *'the "timbu" has a naked tail and the "ticaca" is black, with white stripes on its back, which are not present on the "timbu's" back'*.

Several studies focusing the folk taxonomy have reported that Indian people and fishermen, besides using morphological classification criteria, also use, for example, ecological and behavioural criteria for denominating and identifying animal groups [34,36,22]. In studies on the avifauna classification used by the Wayampi, an Amazonian native people of Brazil, it was observed the preponderance of such criteria, added up of characteristics from their religious belief [30].

Cultural and biological diversity are partly responsible for the richness of names recorded in the folk taxonomy. This is one of the reasons concerning the importance of ethnobiological studies of the denominations that local human populations give to the flora and fauna. This makes easier our understanding on human/Nature relations and can be useful for testing the possible effects of cultural evolution for maintaining certain denominations [22]. Fishermen in the State of Alagoas use fishing nomenclature composed of synonymies, emergent names, and the most commonly used names [8]. The emergence and localization of ichythyonymic denominations may be used as an evidence of cultural evolution of fishermen communities, since they provide variations for a further selection by their peers. Ecological history makes use of the study of changes in the environmental language to infer that history, not evolutionary events, is responsible for the chief changes in the relations between the human society and its close environment [45].

### The use of mastofauna

Food is not simply implicit in the category denominated 'animals', say the inland hunters, who consider as such, just the 'hunting animals'. These are: nine-banded armadillo (*D. novemcinctus*), six-banded armadillo (*E. sexcinctus*), Spix's yellow-toothed cavy (*G. spixii*), and rock cavy (*K. rupestris*), which are also used for other purposes. The armadillos are their favourite generic folk, being the nine-banded armadillo's meat the most appreciated by the hunters. Such preference is confirmed in other northeastern Brazilian states [46], like in Alagoas where 100% of hunters elected this animal's meat as the one with the best pleasant taste, being then one of the most hunted animals [47]. In the Paraiban interland the rock cavy is used as food and its curd is utilized for making cheese.

All the other animals are of secondary importance for them, being only used if captured by the hunters. Cats' furs for example, are only used as ornament; and other animals can be caught and kept in captivity as pets.

The traditional populations of the Paraiban interland also use local faunistic resources for medical purposes in the same way they use the phytotherapeutical resources [48]. When asked about that, the hunters said the generic folk Spix's yellow-toothed cavy (*G. spixii*), rock cavy (*K. rupestris*), and skunk (Didelphidae) are the most hunted animals, whose meat and its broth are used for treating rheumatism, weakness, and loss of appetite.

### The hunter's collection techniques and their implications

The hunters themselves report that these are sporting and subsistence hunting, and for both kinds they utilize shotgun during the day for capturing mammals, birds, and reptiles. During the night they make use of dogs for capturing armadillos and skunk. A hunter said: '*... we arrive at the hill around 10 pm; we loose the dogs and sit down waiting for them; they come back and we move to another place walking for 10 or 15 minutes; the dogs find the animal's burrow and we dig the animal out and capture it*'.

The interviewers report that they do not like to hunt from December to March because of the females' reproductive period or because they are caring after their pups. The hunters said: '*We do not hunt in December and January because the females are pregnant; and we do not hunt during the pups' birth period because if we kill the pregnant female we would kill "one plus four individuals"; and if we kill an animal from January to March, we would kill one or more individuals because the poor pups are in the cave waiting that their parents feed them*'.

This is the way the hunters have to manage their local animal resources, by sparing the females and their pups' lives. However, hunting and deforestation are still the main factors threatening those animal species in the wild, in the region we studied. That paradox suggests a deeper investigation, where can generate quantitative informations about the abundance of the resources mastofaunistics and of the respective hunt products. Like this, showing to real relation resource/hunt of the area.
